# Reproductive characteristics of American bullfrogs (*Lithobates catesbeianus*) in their invasive range of the Pacific Northwest, USA

**DOI:** 10.1038/s41598-020-73206-w

**Published:** 2020-10-01

**Authors:** Jenny Urbina, Evan M. Bredeweg, Christopher Cousins, Andrew R. Blaustein, Tiffany S. Garcia

**Affiliations:** 1 Environmental Sciences Graduate Program, Oregon State University, Corvallis, OR 97331 USA; 2 Department of Fisheries and Wildlife, Oregon State University, Corvallis, OR 97331 USA; 3 Department of Integrative Biology, Oregon State University, Corvallis, OR 97331 USA

**Keywords:** Ecology, Evolution, Zoology, Ecology, Environmental sciences

## Abstract

Invasive species pose a major threat to global biodiversity. The effects of invasive species can be strongly influenced and potentially mediated by their reproductive characteristics, such as fecundity, egg production, and duration and number of reproductive events. Selection for smaller body size at first reproduction can also play a role in their establishment, facilitating colonization and spread. The American bullfrog, native to the eastern U.S. (*Lithobates catesbeianus*), is a species that has invaded more than 40 countries across 4 continents. This species has become especially prevalent in the western United States since its introduction in the early 1900s. This study characterized reproductive characteristics of bullfrogs with emphasis on the minimum size at which males and females reach sexual maturity in the Willamette Valley, Oregon, USA invasion range. We collected and dissected 121 individuals in 2013 and 2017, quantifying characteristics of sexual maturity including snout-vent length, total length, sex, tympanum diameter, presence of distended oviducts or eggs for females, and testes length and sperm activity in males. Our results showed that the minimum reproductive size of both males and females was smaller relative to bullfrogs in their native range as well as in populations across their invasive range. Reduction in size at reproductive maturity is likely impacting the invasive success of American bullfrogs and this study gives us insight on management actions to control the invasion. Applying this insight, managers can adjust their definition of reproductively active adults, increasing the target population of culling and other control methods.

## Introduction

Biological invasions are a significant driver of global change in biodiversity^1,2^. Intentional and unintentional species introductions can result in degraded ecosystem function^3,4^, changes in interspecific interactions^5,6^ and native population declines^7,8^. In addition to environmental impacts, changes to economic growth (i.e. agriculture^9^, and human health^10^) make invasive species one of the costlier disturbances on a global scale^9,11,12^. However, only a relatively small proportion of exotic species succeed in establishing populations within novel regions^13^ or, after becoming established, directly impact invaded ecosystems^14^. Therefore, it is important to understand and evaluate the potential of introduced species to successfully establish. Yet, predicting invasiveness can be difficult as biotic and abiotic factors both play a role determining the establishment of exotic populations^15^.

Trait-based inquiry can be useful when characterizing biological invaders^16^. These include life history characteristics such as growth and reproduction rates, home range size, and diet breadth^17^. Although the strategies by which invasive species establish and spread vary, reproductive traits, such as average clutch size and size at first reproduction can disproportionally affect population dynamics^18^. In particular, invasion potential can be strongly impacted by body size at first reproduction. For example, lionfish (*Pterois* spp) and the brown tree snake (*Boiga irregularis*) have larger body sizes in their invaded ranges, resulting in an increase in the number of offspring released (i.e. propagule pressure)^19,20^. As such, species-specific information on the relationship between body size and reproductive capacity can be useful for management actions that target invasive species^21^.

The unprecedented loss of amphibian biodiversity on a global scale^22,23^ contrasts with the fact that several frog species are successful invaders^24–30^. For example, American bullfrogs (*Lithobates catesbeianus*) have established in over 40 countries across 4 continents and been implicated in the decline of native species across multiple taxonomic groups^28,31,32^. Trait-based research has largely attributed successful bullfrog invasions to initial propagule pressure and biotic tolerance to varying climate regimes^31,33–36^. The ability to reproduce at a smaller body size improves invasion and the range expansion potential of a newly established bullfrog population^37–39^. For example, in Brazil, established populations of American bullfrogs reproduce when males reach 7.6 cm and females reach 6.5 cm snout-vent length (SVL)^40,41^, which is smaller than the minimum sizes at reproduction of 8.5 cm and 12.3 cm in the eastern and western edges of the bullfrog’s native ranges, respectively^34^. Alternatively, Govindarajulu et al.^34^ reported reproductive sizes larger than those found in the northern extent of the bullfrog’s Pacific Northwest range relative to native populations at similar latitudes^42,43^.

We evaluated the size at first reproduction in bullfrogs in the southern extent of their Pacific Northwest USA invaded range and compared with other studies of invaded and native populations of this species. Bullfrogs have become densely populated throughout the region’s low-elevation freshwater habitats after being introduced during the early 1900s to establish farms for exporting frogs to international markets^44^. In areas where they have been introduced, bullfrogs exist without the presence of their native predators, the lack of which can reduce the number of anti-predator responses, potentially impacting their development^45^ allowing for substantial energy allocation for growth and reproduction. We hypothesized that the minimum reproductive size in the Willamette Valley would be smaller than their size in populations at similar latitudes within their native range. Further, we predicted minimum reproductive sizes would more closely match those of populations in Brazil than in their native ranges, as a reduction in size at reproduction could positively affect the ability of bullfrogs to successfully invade new territory, and the lack of native predators in both areas could result in similar developmental patterns despite different abiotic conditions^41,45^. Observing smaller reproductive sizes in both Brazil and the Pacific Northwest USA support bullfrogs as highly suited to colonizing habitat under a wide range of environmental conditions, congruent with their observed spread throughout the globe.

## Methods

We collected American bullfrogs (*Lithobates catesbeianus*) from six locations in Lane (43° 57′ 39.5994″ N, 122° 39′ 42.4794″ W) and Benton County (44° 37′ 41.5194″ N, 123° 23′ 14.6394″ W) (Oregon) where no eradication programs have been established. We sampled 4 ponds with no resident fish populations and two permanent ponds with fish populations (Table 1). Using Visual Encounter Surveys^46^, we sampled and collected metamorphosed bullfrogs during spring and summer breeding seasons of 2013 and 2017 for 150 h. Bullfrogs with a maximum size of approximately 9 cm SVL were targeted, as 8.3 cm is the lowest reported minimum size of reproductive individuals throughout their native range^34^. We collected individuals in this size (~ 9 cm) or that fell below this size. Individuals were transported to Oregon State University where they were euthanized using MS-222 and preserved in 90% ethanol. We followed all institutional and national guidelines for the care and use of animals. This study was approved by the Oregon State University- Institutional Animal Care and Use Committee review board.

**Table 1:** Sampled locations for American bullfrogs (*Lithobates catesbeianus*) in the Willamette Valley.

Location	Coordinates	Fish presence (Yes = Y/No = N)	Hydroperiod
William L. Finley National Wildlife refuge—Lower 22	44° 24′ 47.0" N 123° 19′ 38.0" W	N	Mostly permanent, dry by management
LCC wetlands	44° 00′ 49.5" N 123° 02′ 22.1" W	N	Permanent
Timberline	44° 01′ 13.07" N 123° 08′ 52.07" W	N	Permanent
Barger	44° 04′ 35.8" N 123° 12′ 14.7" W	N	Permanent
William L. Finley National wildlife refuge—Cattail pond	44° 24′ 05.0" N 123° 19′ 27.8" W	Y	Mostly permanent, dry by management
Green Island	44° 08′ 23.6" N 123° 06′ 14.4" W	Y	Permanent

Determination of sexual maturity is more rigorously done by examination of the gonads^34^, as relying only on secondary sexual characteristics can be problematic. Yellow throat coloration and swollen nuptial pads in males are indicators of sexual maturity, but are only present in males. Further, gender differences in tympanum size are not obvious in young individuals. As such, we determined the stage of gonad development for both males and females in addition to measured snout-vent length (SVL), total body length, eye and tympanum diameter, and determined body mass for each individual. For males, we excised, measured and weighed testes with a precision of 0.001 g for mass (Ohaus Adventurer Pro, Pine Brook, NJ USA) and 0.01 mm for length (Marathon, New Brunswick, NJ USA). The right gonad was macerated in 0.5 ml of Holtfreter’s solution 10% to count actively motile sperm using a hemocytometer^47^ while the left gonad was preserved in 90% ethanol for microscopic analysis^48^. Individuals with actively motile sperm were considered reproductively capable. We calculated the male gonadosomatic index (GSI) as GSI = GM × 100/BM, where GM represents gonad mass and BM represents body mass. The value obtained for the right gonad was multiplied by two following Costa et al.^48^. For females, ovarian maturation stages were described following the protocol developed in Costa et al.^49^. Ovarian maturation in female American bullfrogs was categorized into one of five distinct stages: (1) juvenile with thin ovaries, hyaline to whitish and no oocytes distinguishable; (2) beginning of maturation with yellowish ovaries and deeper invaginations, oocytes present; (3) intermediate maturation grayish ovaries with pigmented post-vitellogenic oocytes; (4) advanced maturation high proportion of post-vitellogenic oocytes; and (5) spent ovaries: flaccid, with reduced volume and atresic oocytes^49,50^.

The minimum reproductive size was determined as the minimum SVL when females presented convoluted oviducts or ovaries with eggs inside their thoracic cavity (stages ≥ 2). Males minimum reproductive size was the minimum SVL when they exhibited active sperm. We evaluated if body size of adults between the two sexes were different by analyzing the SVL, total length measurement and body mass using a Student’s t-test with a Welch correction. To evaluate the logistic regression accuracy to predict sexual maturity in males and females, we used a ROC (Receiving Operator Characteristic Curve). This representation shows the ability of the logistic regression to correctly classify cases meeting certain condition (sexually maturity) and cases not meeting the condition of interest. The estimated threshold indicates the point at which the prediction for values meeting the condition is optimal; this is the point at which the sum of the false positives and false negatives is the least.

## Results

**Figure 1:**
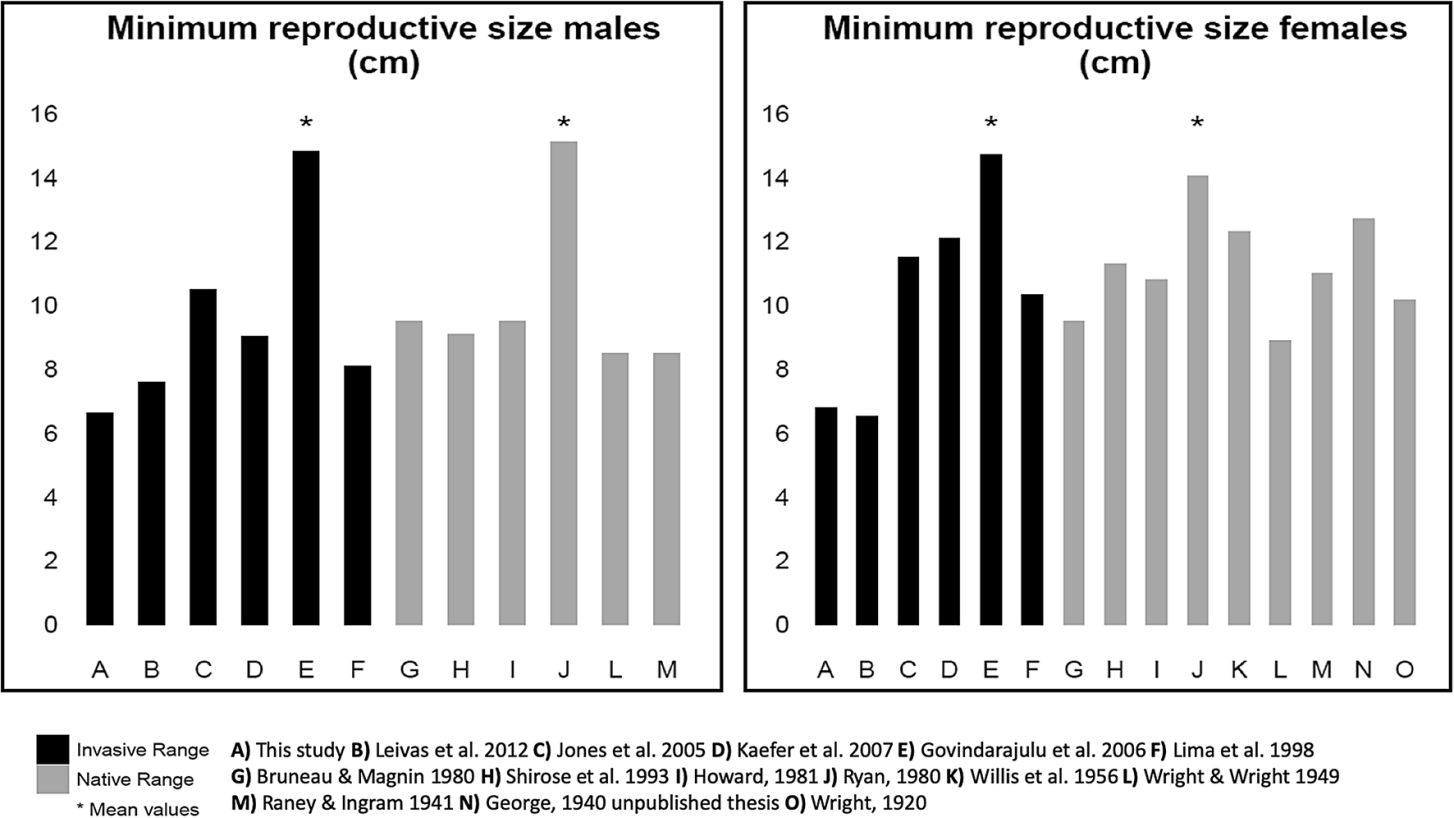
Minimum reproductive size for American bullfrogs (*Lithobates catesbeianus*) in native and invaded ranges of distribution. Measures from reproductive males (left panel) and females (right panel) were compiled from literature.

We collected 121 *L. catesbeianus*: 60 females, 57 males, and 4 individuals that were of undetermined sex. Of these, 22 were reproductively mature adult females and 41 were reproductively mature adult males. In all 60 females captured, including both reproductively mature and immature, SVL varied between 3.8 and 17.6 cm, total length between 8.46 and 34 cm, and body mass ranged from 4.63 to 500 g. In males, SVL varied between 4.01 and 16.5 cm total length between 8.77 and 36 cm, and body mass ranged from 5.37 to 357 g. The minimum reproductive size for females was 6.7 cm and for the males 6.6 cm (Fig. 1). Potentially reproductive males had GSI values between 0.016 and 0.619 with a mean value of 0.147 ± 0.130 SD. For non-reproductive males, the GSI values were between 0.014 and 0.184 with a mean value of 0.049 ± 0.184 SD. Ovarian maturation stages 1 and 2 were found on 38 non-mature females, 16 females were in stage 1, and 22 in stage 2. From the total number of reproductive females (22), 7 females (~ 31%) were at intermediate maturity, 7 females (~ 31%) were at advanced maturity, and 8 (~ 37%) females already reproduced.

**Figure 2:**
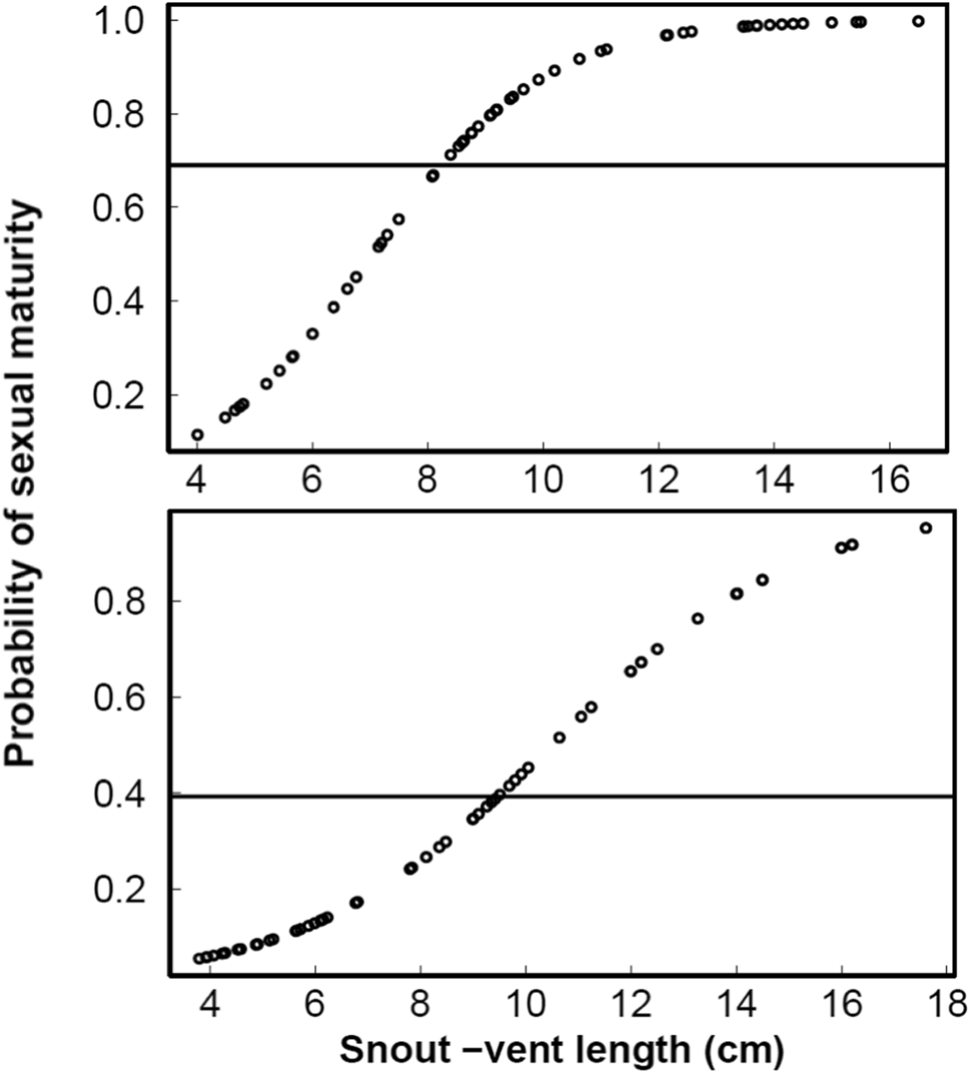
Estimated values for the first maturation of males (top) and females (bottom) of American bullfrog (*L. catesbeianus*) in an invaded range (Willamette Valley, OR). The horizontal line represents the threshold at which males and females are sexually mature.

For mature individuals we did not observe gender differences in size (SVL Welch t-test, t = − 1.22, df = 41.3, and *p* = 0.22; mean ± SD: males SVL = 10.84 ± 2.69 cm, and females SVL = 11.75 ± 2.85 cm), body mass (Welch t-test, t = − 0.97, df = 35.87, and *p* = 0.33; mean ± SD: males BM = 137.93 ± 103 g, and females BM = 169.06 ± 128 g) and total length (Welch t-test, t = − 0.37, df = 46.85, and *p* = 0.71; mean ± SD: males TL = 26.09 ± 5.94 cm, and females TL = 26.65 ± 5.37 cm). Eye diameter was not different between males and females (Welch t-test, t = − 0.78, df = 41.76, and *p* = 0.43; mean ± SD: males ED = 11.91 mm ± 2.73 g, and females ED = 11.31 ± 2.83 mm). On the contrary, tympanum diameter was different between gender (Welch t-test, t = 2.80, df = 59.51, and *p* = 0.006; mean ± SD: males TD = 12.38 ± 4.71 mm, and females TD = 9.83 ± 2.30 mm). The threshold at which ROC curves estimated the accuracy of the logistic regression to predict sexual maturity in males and females as optimal was 0.69 and 0.39 respectively. The SVL value for these thresholds is around 8 cm in males and 10 cm for females (Fig. 2). At these thresholds, males and females were predicted to be sexually mature, with the minimum number of false positives and false negatives.

## Discussion

We found that the minimum reproductive size for male and female American bullfrogs in the southern Willamette Valley was 6.6 cm and 6.7 cm respectively, which is smaller relative to populations within the native range at similar latitudes where, according to secondary sexual characteristics, males and females mature at 9.5 cm and 10.8 cm respectively^51^. Our results highlight how using different type of characteristics can be useful to evaluate reproductive status in an invasive species (Fig. 1 and Table 2). Further, the minimal reproductive size in our study is smaller than those found in invasive bullfrog populations in Brazil, South America, where the minimum reproductive size at sexual maturity was 7.6 cm for males and 6.5 cm for females^41^. Minimum reproductive sizes in our study were smaller relative to other invaded ranges in the United States and Canada, including populations from the northern extent of the Pacific Northwest invaded range (Washington and British Columbia; Table 2). This reduction in minimum reproductive size is likely increasing the number of reproductive events for breeding individuals, thus increasing the propagule pressure of invasive populations in Oregon^41^. Reaching sexual maturity at a smaller body size is likely enhancing invasion potential for populations within the Willamette Valley, Oregon, with individuals reaching breeding age before secondary sexual characteristics are present.

**Table 2:** Minimum reproductive size for American bullfrogs (*Lithobates catesbeianus*) in native and invaded ranges of distribution (ND = no data).

Location	Country	Invasive population (Y–N)	Minimum reproductive size males (cm)	Minimum reproductive size females (cm)	References
Oregon	USA	Y	6.61	6.77	This study
State of Parana	Brazil	Y	7.6	6.5	^41^
Washington	USA	Y	10.5	11.5	^52^
State of Rio Grande	Brazil	Y	9.025	12.083	^50^
British Columbia	Canada	Y	Mean 14.8	Mean 14.7	^34^
State of Minas Gerais	Brazil	Y	8.09	10.33	^40^
Quebec	Canada	N	9.5–11	9.5–11	^42^
Ontario	Canada	N	9.1	11.3	^43^
Michigan	USA	N	9.5	10.8	^51^
New Jersey	USA	N	Mean 15.12	Mean 14.03	^78^
Missouri	USA	N	ND	12.3	^79^
ND	USA	N	8.5	8.9	^80^
New York	USA	N	8.5	11	^81^
Louisiana	USA	N	ND	12.7	^82^Thesis unpublished
ND	USA	N	ND	10.16	^83^

The allocation of energy towards reproduction provides advantages to invading species. The reproductive cycle of American bullfrogs in Oregon is mainly restricted to the summer season when individuals congregate in lentic freshwater systems. Critical factors for breeding include calm water and air temperature above 20 °C^52^. In the Willamette Valley, females can lay 6000–20,000 eggs with body size positively correlated with egg number^53^. In warm water, hatching occurs in 2–5 days and tadpoles can take up to 2 years to reach metamorphosis. However, tadpoles from some populations in the Pacific Northwest invaded range can metamorphose less than 4 months after hatching^54,55^. Males and females in the Willamette Valley may therefore reach their minimum reproductive size less than 2 years after metamorphosis. This change in size may be explained by reaching metamorphosis faster, resulting in smaller juvenile body sizes. Metamorphosing at smaller sizes often results in smaller adult sizes and smaller sizes at maturation for ranids^56^. Alternatively, Bredeweg et al.^57^ found that *Rana aurora* tadpoles that spent less time in the water emerged at smaller sizes, but subsequently had greater initial rates of growth. It is possible that with an increase of post-emergence growth rates, the time to sexual maturity decreases.

The allocation of resources to reproductive traits can increase the rate of population growth, affecting dispersal and result in successful establishment^36^. Although our study did not evaluate range expansion in invasive American bullfrogs, modifications in the allocation of resources to reproductive traits can increase population growth, affecting range expansion and resulting in successful establishment of this species^37,50^. Trade-offs between reproduction and dispersal are critical to understanding the spread of invasive species^58,59^, and individual‐based spatial models predict trade-off outcomes. In amphibians, studies on cane toads (*Rhinella marina*) found that individuals at the invasion front allocated resources to phenotypic traits that facilitate their locomotion while individuals from areas previously colonized allocated resources toward reproduction. For example, toads at the invasion front exhibited narrower heads and longer legs, with males exhibiting smaller testes and females reproducing at lower rates than their conspecifics from interior, established populations^30,60,61^. Similarly, a decrease in the allocation of resources to reproduction at the periphery of the colonized range, is being reported in invasive populations of the African clawed frog *Xenopus laevis*^62^ and the southern African toad *Sclerophrys gutturalis*^63^*.*

Life history characteristics that value adult survivorship over reproductive potential can also increase invasion success^64^. This highlights the tradeoff between earlier sexual maturity and smaller juvenile body size in Oregon bullfrog populations^65^. The estimated size thresholds for males and females to be sexually mature were similar to measurements reported in an invaded range from Brazil^41^. The estimated threshold was greater for females (10 cm) which could indicate that the maturity rate for females is delayed relative to males. Similarly, females of the African clawed frog (*Xenopus laevis*), another successful anuran invader, mature 6 months after metamorphosis at only 6.7 cm in length, providing an advantage in their invasive range^66^. We posit that bullfrog females in the Willamette Valley are allocating energy to early maturation instead of growth^67^. Individuals may also be capable of spawning multiple times during a breeding season with this multiple clutching potentially improving the genetic diversity of the invading populations as one female’s eggs can be fertilized by multiple males^68,69^.

American bullfrog populations are widespread in the Willamette Valley (OR, USA)^31,33^. They displace native anurans via predation, competition, and alterations of microhabitat^70^. Interspecific differences in phenology provide a competitive advantage for late-stage bullfrog larvae in comparison to small and early stage larvae of native amphibian species to acquire limited food resources^71^ and adult bullfrogs are gape-limited generalist predators that can prey on other amphibians as well as other taxonomic groups^25^. In Oregon, bullfrogs have been cited as one of the major threats in population declines of native frog species, including the threatened northern red-legged frog (*Rana aurora)* and the extirpated Oregon spotted frog (*Rana pretiosa)* and they can impact other native pond-breeding species in the region^53,72^. Additionally, adult bullfrogs can play a critical role as carriers of pathogens as ranaviruses, the chytrid fungus *Batrachochytrium dendrobatidis* as well as pathogenic bacteria^73–76^. Research to understand traits explaining advantages of an invasive species can guide strategies to prevent expansion of bullfrogs’ distribution into temporary and permanent habitats where native amphibians and bullfrogs can co-occur^55,77^.

Understanding key traits that predict or enhanced invasion success is critical for the implementation of management and control actions^31^. Characterizing the reproductive activity of breeding bullfrog populations in the Willamette Valley in connection with abiotic factors can be critical in managing the establishment of new populations of this species. Our study identified a decrease in the minimum reproductive size of males and females in invasive American bullfrogs in the Willamette Valley relative to native populations. This finding indicates that we need to modify our view of what constitutes a mature bullfrog in the Willamette Valley, and potentially in other invasion ranges. The results of this study will allow managers undertaking removal efforts to ensure that they are targeting all animals that could possibly be reproductively mature. Figure 2 provides the threshold at which males and females are sexually mature and serves as a guide to managers engaged in removal efforts. Although smaller females can have limited reproductive output, the potential for longer reproductive longevity both over ontogeny and within a breeding season could significantly increase the invasion potential of this critical invasive species.
